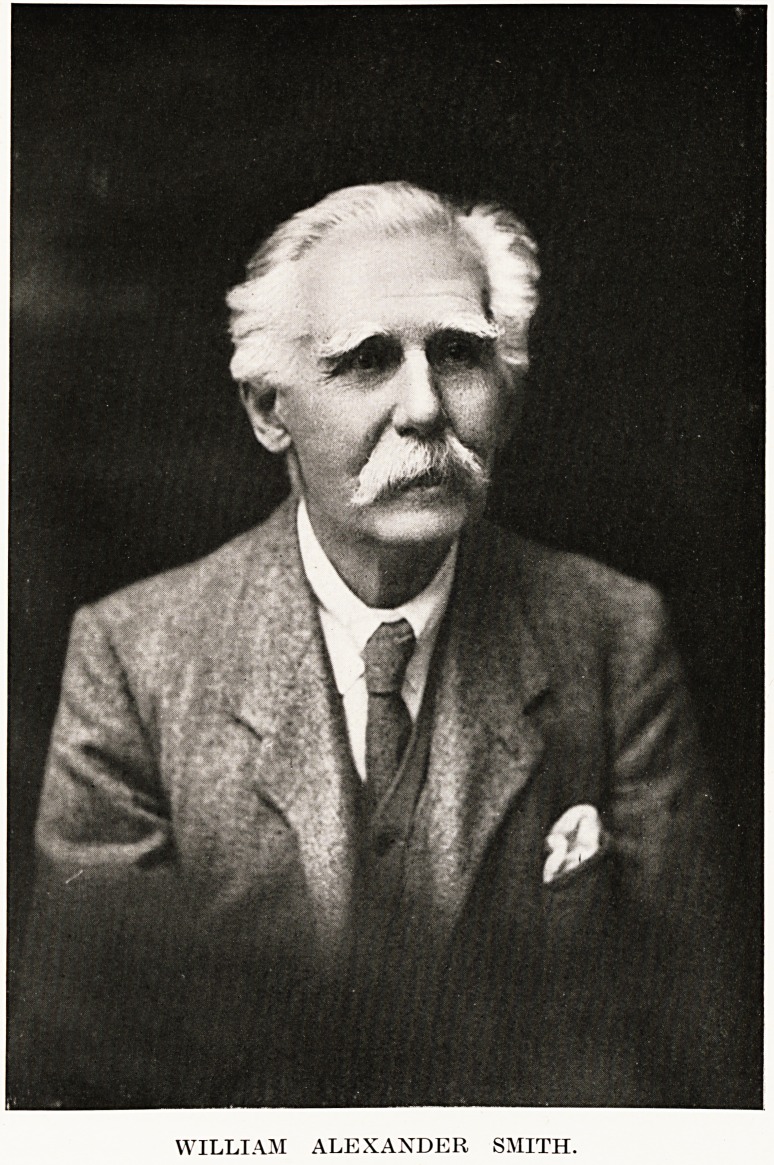# William Alexander Smith

**Published:** 1933

**Authors:** 


### Obituary

### WILLIAM ALEXANDER SMITH, M.A., M.B., M.R.C.S., F.C.S.

Dr. William Alexander Smith was born on August 9th, 1852, at Chesterfield, Derbyshire. His father, Dr. William Smith, after qualifying at the new Bristol School and practising for a time in Park Street and elsewhere, had settled in Chesterfield and been appointed Surgeon to the Hospital. Thus his eldest son "Sandy" came to begin his education in the Chesterfield Grammar School. He next went to a Quaker School under Messrs. Tilden and Smith at Weston-super-Mare. When the father returned to Clifton and built No. 70 Pembroke Road as his home, his son entered Clifton College under Dr. Percival in 1867. He rose to the Sixth, distinguishing himself in chemistry, but also gaining those literary and classical tastes which pervaded all his later life.

On leaving in 1872, he went, after winning an entrance studentship, to Christ Church, Oxford, and finally got First Class Honours in the Natural Science Schools of 1875. At St. Mary's Hospital he took the highest place in the scholarship competition, and qualified in 1880 as M.A., M.B., M.R.C.S., to which he added the F.C.S.

He chose to engage in country practice, and settled down at Newport in Essex, where he was ere long made Surgeon to the Saffron Walden Hospital near there. He had to travel long distances in his dogcart or on horse-back, and thoroughly did he enjoy his open-air life. Even here he got together a little club of friends, a reading society of twelve members, the Duodecimos.

He married Mary Catherine, a daughter of Mr. Mark Whitwill of Bristol, by whom he had one girl, afterwards Mrs. Clover. His wife dying in 1907, he returned to Clifton, practised for a time in Durdham Park, and later on joined his sisters in the family house, 70 Pembroke Road.

When the Panel Referee was called up for war service Dr. Smith was chosen to succeed him, and he did much good

WILLIAM ALEXANDER SMITH.

work of the same kind for the Pensions Board and afterwards under the Insurance officials. He also served this *Journal* for a time during the war as Editorial Secretary.

Besides these duties he had many other interests. He was Secretary of the Shakespeare Society, Chairman of the Research Defence Society, and Treasurer of the Naturalists. Again, besides collecting a marvellous library of his own, he gave much time to helping Mr. C. K. Rudge to discover and sort out the rare volumes among the books of the Medical Library.

When Mr. Rudge died in 1926 he was elected Hon. Medical Librarian. From henceforward he worked assiduously for the Library, searching out and recommending the works needed and making critical lists of the new books and editions with the skill of a booklover and of a ripe scholar.

After five years' service for the Library he was suddenly taken ill at the end of a long day of his referee work. He rallied, but had to give up all his activities. At last, in November, 1932, he moved to Cambridge, to be with his daughter and his grandchildren. For a time he lived happily enough, but a relapse came on and ended fatally on August 3rd, 1933. As a friend described him: "Tall, shaggy, silvery-haired, stooping, uncoated in the coldest weather, with an endearing hesitancy of speech, there was lost to us the figure we loved."

## Figures and Tables

**Figure f1:**